# *Spaced words* and *kmacs*: fast alignment-free sequence comparison based on inexact word matches

**DOI:** 10.1093/nar/gku398

**Published:** 2014-05-14

**Authors:** Sebastian Horwege, Sebastian Lindner, Marcus Boden, Klas Hatje, Martin Kollmar, Chris-André Leimeister, Burkhard Morgenstern

**Affiliations:** 1 University of Göttingen, Institute of Microbiology and Genetics, Department of Bioinformatics, Goldschmidtstraße 1, 37073 Göttingen, Germany; 2 Max-Planck-Institute for Biophysical Chemistry, Department of NMR-based Structural Biology, Group Systems Biology of Motor Proteins, Am Fassberg 11, 37077 Göttingen, Germany; 3 Université d’Évry Val d'Essonne, Laboratoire Statistique et Génome, UMR CNRS 8071, USC INRA, 23 Boulevard de France, 91037 Évry, France

## Abstract

In this article, we present a user-friendly web interface for two alignment-free sequence-comparison methods that we recently developed. Most alignment-free methods rely on *exact* word matches to estimate pairwise similarities or distances between the input sequences. By contrast, our new algorithms are based on *inexact* word matches. The first of these approaches uses the relative frequencies of so-called *spaced words* in the input sequences, i.e. words containing ‘don't care’ or ‘wildcard’ symbols at certain pre-defined positions. Various distance measures can then be defined on sequences based on their different spaced-word composition. Our second approach defines the distance between two sequences by estimating for each position in the first sequence the length of the longest substring at this position that also occurs in the second sequence with up to *k* mismatches. Both approaches take a set of deoxyribonucleic acid (DNA) or protein sequences as input and return a matrix of pairwise distance values that can be used as a starting point for clustering algorithms or distance-based phylogeny reconstruction. The two alignment-free programmes are accessible through a web interface at ‘Göttingen Bioinformatics Compute Server (GOBICS)’: http://spaced.gobics.de http://kmacs.gobics.de and the source codes can be downloaded.

## INTRODUCTION

Comparative sequence analysis and phylogeny reconstruction are traditionally based on pairwise or multiple sequence alignments. These classical approaches are well-established and regarded as standard methods of sequence analysis. There are, however, various limitations associated with these approaches if large data sets are to be analysed. Aligning whole genomes of higher eukaryotes requires excessive computation time and the reliability of such global sequence alignments is often limited. Moreover, because of genome duplications and rearrangements it is often not possible to align entire genomes, so alignable homologous portions of the genomes under study have to be identified in a first step. With the growing amount of sequence data in public and private databases, produced by next-generation sequencing technologies, there is now a strong demand for faster sequence-analysis algorithms. To overcome the limitations of traditional alignment-based approaches, various alignment-free methods have been developed over the past two decades, see (1) for an overview. The run time of these algorithms is usually proportional to the total length of the input sequences, while a pairwise alignment takes time proportional to the product of the sequence lengths. It is known however, that alignment-free methods are generally less accurate than alignment-based methods.

Most alignment-free methods rely on the frequencies of words of a fixed length *k*, also denoted as *k-mers* or *k-grams*. Various distance measures can be applied to the corresponding frequency vectors to estimate similarities or distances between sequences (2–5). Other methods are based on word matches of variable length (6,7). One established method is the *average common substring approach* (8) that is based on the so-called *matching statistics* (9). Here, for each position *i* in one sequence, one calculates the *longest* substring starting at *i* that also occurs in the second sequence. The *average* length of these longest common substrings is a measure of *similarity* between two sequences and can be turned into a symmetric distance measure. A similar approach was introduced simultaneously as the *shortest unique substrings (shustring) approach* (10). As a further improvement, these authors also derived an estimator for the rate of substitutions between two unaligned sequences depending on the average *shustring* length (11).

All above mentioned methods are based on *exact* word matches. A well-known drawback of using exact word matches in sequence comparison is that word matches at neighbouring sequence positions are statistically far from independent. In database searching, exact word matches have therefore been replaced by so-called *spaced seeds* defined by patterns of *match* and *don't care* positions (12). It has been shown in many studies that such *spaced seeds* are far superior to *contiguous (exact)* word matches in detecting local homologies (12–14). Motivated by this approach, we recently proposed two methods for alignment-free sequence comparison that are based on inexact word matches, *spaced words* (15) and *kmacs* (16). In this paper, we present a user-friendly web interface for these two methods.

## SPACED WORDS

As most alignment-free sequence-analysis programmes, *spaced words* considers the composition of the input sequences in terms of short subsequences of a fixed size. But while most other approaches calculate the relative frequencies of *contiguous* words of a fixed length, our approach uses patterns of *match* and *don't care* positions and calculates the frequencies of *spaced words* according to these patterns. Thus, a *spaced word* over an alphabet Σ can be seen as a word composed of characters from Σ and *wild-card* characters (the alphabet Σ represents the four nucleotides for deoxyribonucleic acid (DNA) sequences or the 20 amino acids for protein sequences). For example*,* the spaced word ‘T**AG*T’ has a ‘*T*’ at positions one and seven, an ‘*A*’ at position four and a ‘*G*’ at position five. Arbitrary nucleotides are possible at positions two, three and six.

The basic version of this *spaced-word* approach uses one single fixed pattern *P* of *match* and *don't care* positions, represented as a sequence of ‘1’ and ‘0’, respectively, and calculates the frequencies of spaced words with respect to this pattern (17). The number of *match* positions in a pattern or spaced word is called its *weight* *k*. Consider, e.g. the sequence

\begin{equation*} S = ATTATGCTAG \end{equation*}

and the pattern *P* = 11001. From left to right, we find in *S* six spaced words

\begin{equation*} AT**T, TT**G, TA**C, AT**T, TG**A, GC**G. \end{equation*}

Thus, the relative frequency of the spaced word ‘AT**T’ in *S* is 2/6, the relative frequencies of ‘TT**G’, ‘TA**C’, ‘TG**A’ and ‘GC**G’ are 1/6 each, and the frequencies of all other spaced words in *S* with respect to the pattern *P* are 0.

After calculating the relative frequencies of all spaced words according to the fixed pattern *P*, our programme can use different distance measures to define pairwise distances among the input sequences based on their relative spaced-word frequencies. Currently, we are using the *Euclidean* and the *Jensen-Shannon (JS)* (18) distance metrics.

In a recent paper, we proposed a fast implementation of this approach using recursive hashing and bit operations (15). This allowed us to extend our approach by using a whole set $\mathcal {P}$ of patterns instead of one single pattern to define spaced words. To define a distance between two sequences, the programme *averages* the distances calculated with respect to all individual patterns in the set $\mathcal {P}$. We showed that this *multiple-pattern* approach leads to better and statistically more stable results.

## KMACS: THE *k*-MISMATCH AVERAGE COMMON SUBSTRING APPROACH

While our *spaced-words* approach defines distances between sequences based on spaced words of a fixed length and is a generalization of the commonly used word-frequency approaches, *kmacs* defines a distance using the average length of *inexact* substrings shared by two sequences. More specifically, *kmacs* estimates for each position *i* in the first sequence the longest substring starting at *i* and matching some substring in the second sequence with up to *k* mismatches. It defines the average of these values as a measure of similarity between the sequences and turns this into a symmetric distance measure, see (16) for details. This is a generalization of the *average common substring* approach (8). A fast implementation of this approach using *generalized enhanced suffix arrays* (19) has been described in (16).

## INPUT

**Figure 1.**
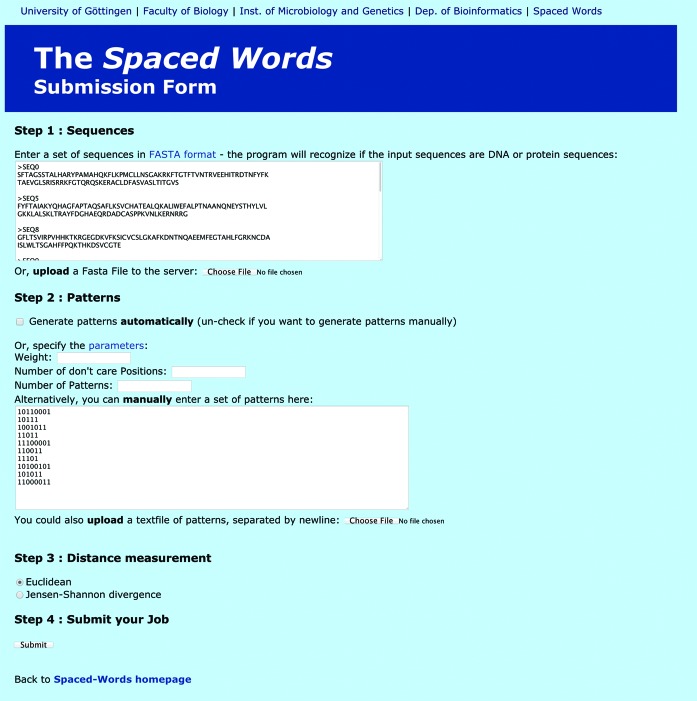
Submission page for *spaced words*. The user can upload or copy-paste input sequences in FASTA format. A set $\mathcal {P}$ of patterns can be generated (i) randomly with default parameters, (ii) randomly with user-defined values for *weight,* i.e. number of *match* positions, number of *don't care* positions and number of patterns in $\mathcal {P}$ or (iii) a pre-defined set of patterns can be uploaded or copy-pasted. The user can chose between the *Euclidean* distance and the *Jensen-Shannon* distance to estimate distances between the input sequences based on their spaced-word frequencies.

For both programmes, the main input is a set of two or more DNA or protein sequences in FASTA format. At our web server, the input sequences can be either uploaded as a single file or pasted into a window; the submission page for *spaced words* is shown in Figure 1. On the web server, the total length of the input sequences is limited to 10 *mb* and 500 sequences. For the downloadable programme code, there are no such limitations but a warning is given if a programme run is expected to use too much of the main memory on the user's computer.

For *spaced words*, three different options are provided to create a set $\mathcal {P}$ of patterns. (i) $\mathcal {P}$ can be generated automatically by our software. In this case, 10 random patterns are generated using default values for the *weight* (number of *match* positions) and the *length* of the patterns, depending on the length of the input sequences. (ii) Alternatively, the user can specify values for *weight* and *length* as well as for the number of patterns in $\mathcal {P}$. The programme then generates a set of random patterns according to these parameters. (iii) Finally, the user can upload a pre-defined set of patterns in a format specified on the web site. Note that with options (i) and (ii), all generated patterns in $\mathcal {P}$ will have the same *weight* and *length* (and therefore also the same number of ‘don't care’ positions). With option (iii) however, it is possible to use patterns of different weight or length.

Our web server offers the *Euclidean* distance and, alternatively, the *Jensen-Shannon* distance to calculate pairwise distances between the input sequences based on their spaced-word frequencies with respect to the specified set $\mathcal {P}$ of patterns. We are planning to provide further distances metrics that can be used as an alternative to those two distances.

For *kmacs*, the only parameter to be chosen by the user is the number *k* of mismatches; values between *k* = 0 and *k* = 100 can be selected, the default value is *k* = 5. With *k* = 0, *kmacs* corresponds to the previously published *average common substring* approach (8).

## OUTPUT

**Figure 2.**
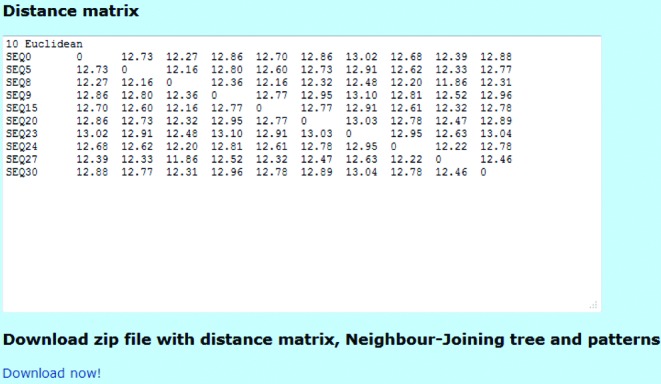
A matrix with pairwise distances of the input sequences is generated by our server. The distance matrix can be downloaded together with a *Neighbour-Joining* tree generated from these distances and, for *spaced words*, with the set $\mathcal {P}$ of patterns that was used in the programme run.

For both programmes, *spaced words* and *kmacs*, the output is a matrix of pairwise distance values in *Phylip* format, as shown in Figure 2. In addition, a *Neighbour-Joining* tree calculated from this distance matrix is provided in *Newick* format and as a graphical representation. For *spaced words*, the set $\mathcal {P}$ of patterns that was used in the programme run is provided.

## TEST RESULTS

In (15) and (16) we evaluated *spaced words* and *kmacs* extensively on real and simulated benchmark sequences. We constructed phylogenetic trees using the distance values calculated by our new software and other alignment-free methods and compared these trees to trusted reference trees. Here, we could show that, on most of our test examples, our new methods were superior to existing approaches to alignment-free sequence comparison. In some cases the quality of trees constructed with our new alignment-free methods was even superior to trees constructed with a slow but generally more accurate approach based on multiple sequence alignment.

As shown in (15), the results of *spaced words* are improved if the number of patterns is increased, but this also increases the programme run time. On simulated DNA and protein sequences, we observed that the quality of the results converges to an optimum between 50 and 70 patterns. In the interest of programme run time, however, the default value on our web server is 10 patterns. For *kmacs*, we found that the programme produces good results on our simulated sequence sets with values of *k* > 10. Further increasing *k* improved the results slightly, but slowed down the programme. On real-world benchmark sequences the results were similar but less regular (16). In the interest of programme run time, the default value on our web server is *k* = 5. When comparing *spaced words* and *kmacs*, we observed that *kmacs* performs slightly better than *spaced words* on protein sequences, while on genomic sequences *spaced words* seems to be superior (16). On protein sequences, *spaced words* produced better phylogenies when used with the *Jensen-Shannon* distance than with the *Euclidean* distance. On genomic sequences, however, the *Euclidean* distance led sometimes to better results.

**Figure 3.**
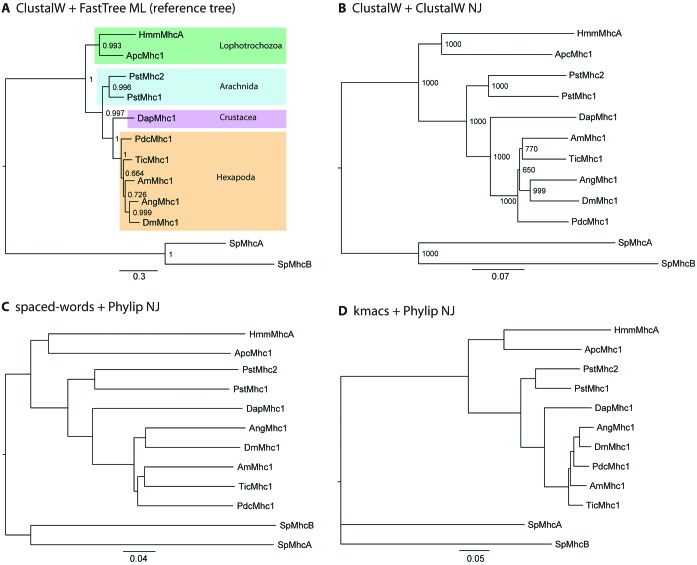
Phylogenetic trees of ten bilaterian muscle myosin heavy chain proteins and two non-muscle myosin heavy chain proteins from *Schizosaccharomyces pombe* (Sp), which were used as outgroup. (**A**) Maximum-likelihood topology generated under the JTT model (22) in FastTree v. 2.1 (23) Support for all branchings is given as likelihood bootstrap values. The 12 myosins were aligned with *Clustal W* (24) using standard parameters. (**B**): *Neighbour Joining* tree of the 12 myosins generated with *Clustal W* with 1000 bootstraps. (**C** and **D**) *Neighbour Joining* trees constructed with Phylip (25) based on the distance matrices generated with *spaced words* and *kmacs*, respectively. Both programmes were used through our web interface with default parameters. Species abbreviations are: Hmm: *Hymenolepsis microstoma*; Apc: *Aplysia californica*; Pst: *Parasteatoda tepidariorum*; Dap: *Daphnia pulex*; Pdc: *Pediculus humanus corporis*; Tic: *Tribolium castaneum*; Am: *Apis mellifera*; Ang: *Anopheles gambiae*; Dm: *Drosophila melanogaster*.

Figure 3 shows phylogenetic trees constructed from ten bilaterian muscle myosin heavy chain proteins and two non-muscle myosin heavy chain proteins from *Schizosaccharomyces pombe* (Sp), which were used as outgroup. In order to assess the trees that we calculated from the distance matrices generated with *spaced words* and *kmacs*, we calculated a reference tree using the *Maximum-Likelihood* method based on the aligned sequences. The reference tree (Figure 3A) is in accordance with the phylogeny of the respective species as discussed in (20). A tree calculated with *Neighbour Joining* from the aligned sequences only differs in the grouping of the *Apis mellifera* myosin (AmMhc1; Figure 3B). While the bootstrap support is high for all other branchings, the support for the grouping of *A. mellifera* and *Tribolium castaneum* (TicMhc1) is relatively low indicating possible alternative branchings. The branchings in the same region are also affected in the *spaced-words* (Figure 3C) and *kmacs* based trees (Figure 3D). While in the *spaced-words* tree the grouping of the Diptera containing the *Anopheles gambiae* (AngMhc1) and *Drosophila melanogaster* (DmMhc1) sequences is affected, the branching of the *Pediculus humanus corporis* (PdcMhc1) myosin is wrong in the *kmacs* tree. However, the major subgroups of the insects diverged in a relatively short time span leading to short distances of the respective branchings and wrong groupings in many insect trees (21). Overall, the relative distances of the sequences are remarkably accurate in the trees that we calculated with our new alignment-free methods. For example, HmmMhcA is always more divergent than ApcMhc1, and PstMhc2 is slightly more divergent than PstMhc1. Because it is common practise in molecular systematics to use alternative methods as each method of phylogenetic inference has assumptions and advantages/disadvantages, the *spaced-words* and *kmacs* approach could be a valuable extension to the repertoire of phylogenetic reconstruction methods.

## DISCUSSION

In our previous publications, we showed that *spaced words* and *kmacs* are fast methods for sequence comparison that can be used to estimate phylogenetic distances between DNA and protein sequences. Trees constructed from these distances are generally of high quality. The software that we provide through our web interface and as downloadable source code is based on advanced string-comparison algorithms.

The idea of using inexact word matches for alignment-free sequence comparison in this way is new, and various further developments are thinkable. For example, we currently provide two distance measures based on spaced-word frequencies, the *Jensen-Shannon* distance and the classical *Euclidean* distance. In our test runs, these metrics produced reasonable results, but both of them rely on *ad hoc* definitions, without any statistical model behind them, as is typical in alignment-free sequence comparison. To our knowledge, the only alignment-free approach that estimates evolutionary distances based on an explicit stochastic model is *K*_*r*_ (11). It seems worthwhile to explore other distance metrics in the context of *spaced words* and to estimate evolutionary distances using models of molecular evolution and results on word frequencies (26). Similarly, it would be interesting to establish a mathematical relation between the average length of the *k*-mismatch substrings in our programme *kmacs* and the ‘true’ evolutionary distance between sequences according to a stochastic model.

Finally, spaced-word frequencies and *k*-mismatch substrings can be used not only to estimate phylogenetic distances, but also as a basis for supervised or unsupervised clustering and classification methods. We will explore these approaches and will add the corresponding functionalities to our web server in the future.

## References

[1] Vinga S., Almeida J. (2003). Alignment-free sequence comparison—a review. Bioinformatics.

[2] Chor B., Horn D., Levy Y., Goldman N., Massingham T. (2009). Genomic DNA k-mer spectra: models and modalities. Genome Biol..

[3] Höhl M., Rigoutsos I., Ragan M.A. (2006). Pattern-based phylogenetic distance estimation and tree reconstruction. Evol. Bioinform. Online.

[4] Vinga S., Carvalho A.M., Francisco A.P., Russo L.M.S., Almeida J.S. (2012). Pattern matching through Chaos Game Representation: bridging numerical and discrete data structures for biological sequence analysis. Algorithms Mol. Biol..

[5] Sims G.E., Jun S.-R., Wu G.A., Kim S.-H. (2009). Alignment-free genome comparison with feature frequency profiles (FFP) and optimal resolutions. Proc. Natl. Acad. Sci. U.S.A..

[6] Comin M., Verzotto D. (2012). Alignment-free phylogeny of whole genomes using underlying subwords. Algorithms Mol. Biol..

[7] Didier G., Corel E., Laprevotte I., Grossmann A., Landés-Devauchelle C. (2012). Variable length local decoding and alignment-free sequence comparison. Theor. Comput. Sci..

[8] Ulitsky I., Burstein D., Tuller T., Chor B. (2006). The average common substring approach to phylogenomic reconstruction. J. Comput. Biol..

[9] Gusfield D. (1997). Algorithms on Strings, Trees, and Sequences: Computer Science and Computational Biology.

[10] Haubold B., Pierstorff N., Möller F., Wiehe T. (2005). Genome comparison without alignment using shortest unique substrings. BMC Bioinformatics.

[11] Haubold B., Pfaffelhuber P., Domazet-Loso M., Wiehe T. (2009). Estimating mutation distances from unaligned genomes. J. Comput. Biol..

[12] Ma B., Tromp J., Li M. (2002). PatternHunter: faster and more sensitive homology search. Bioinformatics.

[13] Burkhardt S., Kärkkäinen J. (2003). Better filtering with Gapped q-Grams. Fundam. Inf..

[14] Keich U., Li M., Ma B., Tromp J. (2004). On spaced seeds for similarity search. Discrete Appl. Math..

[15] Leimeister C.-A., Boden M., Horwege S., Lindner S., Morgenstern B. (2014). Fast alignment-free sequence comparison using spaced-word frequencies. Bioinformatics.

[16] Leimeister C.-A., Morgenstern B. (2014). kmacs: the k-mismatch average common substring approach to alignment-free sequence comparison.

[17] Boden M., Schöneich M., Horwege S., Lindner S., Leimeister C.-A., Morgenstern B. (2013). Alignment-free sequence comparison with spaced *k*-mers.

[18] Lin J. (1991). Divergence measures based on the Shannon entropy. IEEE Trans. Inform. Theory.

[19] Fischer J. (2011). Inducing the LCP-Array.

[20] Kollmar M., Hatje K. (2014). Shared gene structures and clusters of mutually exclusive spliced exons within the metazoan muscle myosin heavy chain genes. PLoS One.

[21] Odronitz F., Becker S., Kollmar M. (2009). Reconstructing the phylogeny of 21 completely sequenced arthropod species based on their motor proteins. BMC Genomics.

[22] Jones D., Taylor W., Thornton J. (1992). The rapid generation of mutation data matrices from protein sequences. CABIOS.

[23] Price M.N., Dehal P.S., Arkin A.P. (2010). FastTree 2 approximately maximum-likelihood trees for large alignments. PLoS One.

[24] Chenna R., Sugawara H., Koike T., Lopez R., Gibson T.J., Higgins D.G., Thompson J.D. (2003). Multiple sequence alignment with the Clustal series of programs. Nucleic Acids Res..

[25] Felsenstein J. (1989). PHYLIP - Phylogeny Inference Package (Version 3.2). Cladistics.

[26] Robin S., Rodolphe F., Schbath S. (2005). DNA, Words and Models: Statistics of Exceptional Words.

